# Neurofibromin regulates metabolic rate via neuronal mechanisms in *Drosophila*

**DOI:** 10.1038/s41467-021-24505-x

**Published:** 2021-07-13

**Authors:** Valentina Botero, Bethany A. Stanhope, Elizabeth B. Brown, Eliza C. Grenci, Tamara Boto, Scarlet J. Park, Lanikea B. King, Keith R. Murphy, Kenneth J. Colodner, James A. Walker, Alex C. Keene, William W. Ja, Seth M. Tomchik

**Affiliations:** 1 Department of Neuroscience, The Scripps Research Institute, Scripps Florida, Jupiter, FL USA; 2 Department of Biological Sciences, Florida Atlantic University, Jupiter, FL USA; 3 Program in Neuroscience and Behavior, Mount Holyoke College, South Hadley, MA USA; 4 Center for Genomic Medicine, Massachusetts General Hospital, Harvard Medical School, Boston, MA USA; 5 Cancer Program, Broad Institute of MIT and Harvard, Cambridge, MA USA; 6 Present Address: Department of Physiology, Trinity College Institute of Neuroscience, Trinity College Dublin, Dublin, Ireland

**Keywords:** Neuroscience, Diseases of the nervous system, Neural circuits, Fat metabolism, Feeding behaviour

## Abstract

Neurofibromatosis type 1 is a chronic multisystemic genetic disorder that results from loss of function in the neurofibromin protein. Neurofibromin may regulate metabolism, though the underlying mechanisms remain largely unknown. Here we show that neurofibromin regulates metabolic homeostasis in *Drosophila* via a discrete neuronal circuit. Loss of neurofibromin increases metabolic rate via a Ras GAP-related domain-dependent mechanism, increases feeding homeostatically, and alters lipid stores and turnover kinetics. The increase in metabolic rate is independent of locomotor activity, and maps to a sparse subset of neurons. Stimulating these neurons increases metabolic rate, linking their dynamic activity state to metabolism over short time scales. Our results indicate that neurofibromin regulates metabolic rate via neuronal mechanisms, suggest that cellular and systemic metabolic alterations may represent a pathophysiological mechanism in neurofibromatosis type 1, and provide a platform for investigating the cellular role of neurofibromin in metabolic homeostasis.

## Abstract (web summary)

Neurofibromatosis type 1 (NF1) is a genetic disorder caused by mutations in neurofibromin and associated with disruptions in physiology and behavior. Here the authors show that neurofibromin regulates metabolic homeostasis via a discrete brain circuit in a Drosophila model of NF1.

## Introduction

Neurofibromatosis type 1 (NF1) is a monogenetic disorder affecting 1 in ~3500 individuals. Caused by mutations in the *NF1* gene, this disorder is characterized by benign tumors of the nervous system called neurofibromas, as well as increased susceptibility to a range of complications, including various cancers and neurocognitive deficits (e.g., attention-deficit/hyperactivity disorder, autism spectrum disorder, visuospatial memory impairments)^1,2^. The *NF1* tumor suppressor gene encodes a large protein called neurofibromin (Nf1), which contains a central GAP-related domain (GRD) that enhances the GTPase activity of the small guanine nucleotide binding protein Ras, thereby down-regulating its biological activity^3,4^. Ras, in turn, signals through multiple effectors, including mTOR, ERK, and potentially cAMP/PKA (indirectly)^5^. Due to the numerous cellular functions of Ras, as well as interactions of Nf1 with other signaling molecules^3,6,7^, loss of Nf1 results in pleiotropic effects on cellular and organismal physiology that drive multisystemic dysfunction. However, the mechanisms through which *NF1* mutations disrupt cellular physiology and behavior remain poorly understood.

Emerging evidence suggests that Nf1 regulates cellular and organismal metabolism. Neurofibromatosis type 1 has been associated with short stature, as well as reduced body mass index in males^8,9^, although the mechanisms underlying these manifestations are unknown. Alterations in certain metabolites have been reported in NF1 patients, though these are sex-specific^8^. The incidence of diabetes mellitus, deaths from diabetes mellitus, and fasting blood glucose levels are lower in individuals with NF1 than in healthy controls^10^. Increased resting energy expenditure has been reported in females with neurofibromatosis type 1^11^. Nf1 haploinsufficient mice exhibit alterations in metabolism, including reduced fat mass, increased glucose clearance and insulin sensitivity, and reduced susceptibility to diet-induced obesity and hyperglycemia^12^. Of potential importance for NF1-associated tumorigenesis, loss of Nf1 increases glycolysis and decreases respiration via Ras/ERK signaling in mitochondria^13^. Therefore, Nf1 regulates various aspects of cellular and organismal metabolism. These alterations in metabolism may contribute to disease pathophysiology, though the underlying mechanisms are not well understood.

Here we investigate the role of the *Drosophila melanogaster NF1* ortholog in metabolic regulation. This provides an excellent model for studying the fundamental biology of NF1 and the cellular/circuit effects associated with loss of Nf1 function. *Drosophila* Nf1 is ~60% identical to the human protein and similarly mediates Ras signaling^14^. Flies with *NF1* mutations exhibit small body size^14–16^, impaired circadian rhythms^17^, learning and memory deficits^18–20^, decreased lifespan via increased susceptibility to oxidative stress^21^, and increased spontaneous grooming^22,23^. These changes demonstrate widespread alterations in cellular/neuronal function, raising the possibility that metabolism could also be altered. Here we report that loss of Nf1 increases metabolic rate, feeding, and energy homeostasis via actions on a central neuronal circuit.

## Results

### Loss of Nf1 increases metabolic rate via neuronal mechanisms

**Fig. 1:**
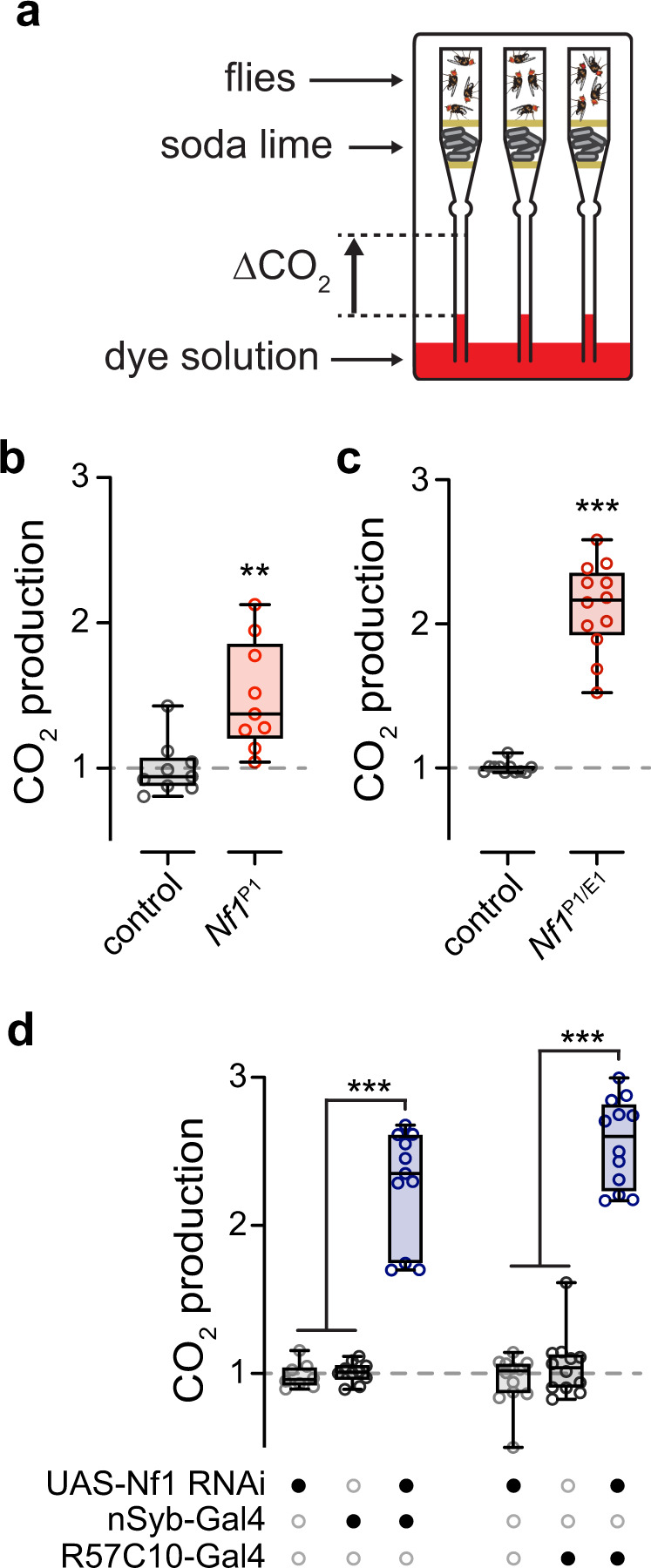
Loss of Nf1 increases CO_2_ production via neuronal mechanisms. **a** Diagram of respirometry apparatus. **b** Normalized CO_2_ production in *Nf1*^P1^ mutants and *w*^CS10^ controls. **p = 0.002 (Mann–Whitney, two-sided; *n* = 9). **c** Normalized CO_2_ production in heteroallelic *Nf1*^*P1*/*E1*^ mutants (*n* = 12) and controls (*n* = 11). ***p < <0.0001 (Mann–Whitney, two-sided). **d** Normalized CO_2_ production when Nf1 was knocked down with RNAi driven by pan-neuronal Gal4 lines: nSyb- and R57C10-Gal4. Gal4/+ and UAS/+  are heterozygous controls. ***p < 0.001 (Dunn’s test, two-sided; for nSyb-Gal4: *n* = 8 UAS-Nf1 RNAi/+, *n* = 10 nSyb-Gal4/+, *n* = 11 experimental group. For R57C10-Gal4: *n* = 12 per genotype). Each “*n*” = single respirometer (containing 4 animals). Box plots—box: 1st to 3rd quartiles, median: line, whiskers: min–max, individual data points: circles.

To initially test whether Nf1 affects metabolic rate, we examined CO_2_ production via respirometry in *Drosophila* (Fig. 1a)^24^. In insects, whole-body CO_2_ production provides a readout of metabolic rate and can be readily measured in freely moving animals^25^. We first compared *Nf1*^P1^ mutants, which harbor a large deletion in the *NF1* locus, including the central catalytic GRD^14^, with *w*^CS10^ controls. *Nf1*^P1^ mutant flies exhibited significantly elevated CO_2_ output compared to controls (Fig. 1b and Supplementary Fig. 1a), suggesting that Nf1 plays a role in metabolic regulation. For additional confirmation, we tested a heteroallelic combination of the *Nf1*^P1^ deletion with a nonsense mutation, *Nf1*^E1^, which results in loss of protein product^16^. *Nf1*^P1/E1^ mutants also exhibited significantly elevated CO_2_ output relative to genotype-matched control flies (Fig. 1c and Supplementary Fig. 1b). To test whether the effect observed in *Nf1* mutants was localized to neurons, we knocked down Nf1 with RNAi using the Gal4/UAS system. In independent experiments, two different pan-neuronal Gal4 drivers were used to drive RNAi: nSyb-Gal4 and R57C10-Gal4. In each case, a significant elevation in CO_2_ production was observed in the experimental group relative to flies harboring either the heterozygous driver (Gal4/+) or effector (UAS/+) transgenes alone (Fig. 1d and Supplementary Fig. 1c). Taken together, these findings suggest that the loss of Nf1 function elevates metabolic rate via neuronal mechanisms.

**Fig. 2:**
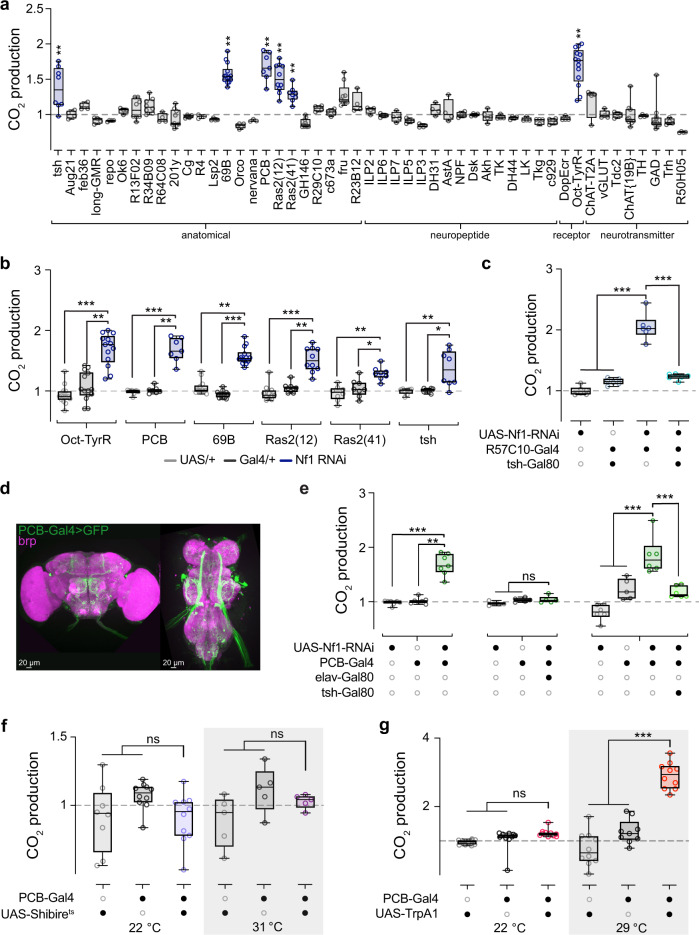
A sparse set of neurons mediates metabolic rate via Nf1. **a** Screen for neuronal subsets in which knocking down Nf1 elevates CO_2_ production. CO_2_ production from each Gal4 line is normalized to its own genetic controls. **b** Significant lines from a, showing RNAi and heterozygous controls. Sample sizes and *p*-values in Source data file for (**a**, **b**). *p < 0.05, **p < 0.01, ***p < 0.001 (Dunn’s test, two-sided; *n* = 7–14). **c** Normalized CO_2_ production, knocking down Nf1 pan-neuronally with R57C10-Gal4, with and without the tsh-Gal80 repressor. ***p < 0.001 (Sidak, two-sided; *n* = 6). **d** Expression of GFP in neurons labeled by the PCB-Gal4 driver. Bruchpilot (brp) counterstains the neuropil. Representative image shown. **e** Normalized CO_2_ production, knocking down Nf1 in neurons with the PCB-Gal4 driver (left) (**p < 0.01 ***p < 0.001 [Dunn’s test, two-sided; *n* = 7]), adding the elav-Gal80 repressor (middle) (experimental *p* = 0.0678 re: UAS-Nf1 RNAi/+, *p* > 0.999 re: PCB-Gal4/+ [Dunn’s test, two-sided; *n* = 5]), or adding the tsh-Gal80 repressor (right) (***p < 0.001 [Sidak, two-sided; *n* = 6]). The left data set is duplicated from (**b**) for comparison. **f** Normalized CO_2_ production when PCB-Gal4 neurons were blocked using UAS-Shibire^ts^ at 31 °C (right) and control temperature (22 °C, left). At 22 °C, experimental *p* > 0.999 re: UAS-Shibire^ts^/+, *p* = 0.147 re: PCB-Gal4/+. (Sidak, two-sided; *n* = 8 UAS-Shibire^ts^/+, *n* = 10 PCB-Gal4/+, and *n* = 10 experimental). At 31 °C experimental *p* = 0.590 re: UAS-Shibire^ts^/+, *p* = 0.903 re: PCB-Gal4/+. (Sidak, two-sided; *n* = 5 per genotype). **g** Normalized CO_2_ production following activation of PCB-Gal4 neurons using UAS-TrpA1 at 29 °C (right) and control temperature (22 °C, left). At 22 °C experimental *p* = 0.269 re: UAS-TrpA1/+, *p* = 0.655 re: PCB-Gal4/+. (Sidak, two-sided; *n* = 10 per genotype). At 29 °C ***p < 0.001 (Sidak, two-sided; *n* = 10 UAS-TrpA1/+, *n* = 9 PCB-Gal4/+, *n* = 10 experimental). For each experiment, data were normalized to the mean of both controls at each temperature. Each “*n*” = single respirometer (containing 4 animals). Box plots—box: 1st to 3rd quartiles, median: line, whiskers: min–max, individual data points: circles. ns: not significant.

To identify the neurons responsible for the increase in CO_2_ production, we knocked down Nf1 using Gal4 drivers that are selective for different populations of neurons. Drivers were selected that express in neurons that release particular neurotransmitters (e.g., cholinergic neurons [ChAT]), modulate growth and/or metabolism (e.g., insulin-like peptides [ILPs], c673a)^26^, or mediate previously characterized Nf1 effects on growth in larvae (e.g., Ras2)^16^. For each line, the experimental group was compared to the two heterozygous genetic controls (Gal4/+ and UAS/+) within experiments. Among 49 lines tested, 6 significantly elevated metabolic rate relative to controls (Fig. 2a, b and Supplementary Fig. 1e). We classified the Gal4 drivers according to whether they label anatomically-defined sets of neurons (“anatomical”), neuropeptidergic classes, neurotransmitters, or neurotransmitter receptors; five of the six positive lines labeled anatomical subsets of neurons (typically spatially-distributed sets). The positive lines were: Oct-TyrR, PCB, 69B, Ras2(12), Ras2(41), and tsh (Fig. 2b and Supplementary Fig. 1e). The Oct-TyrR-Gal4 driver labels subsets of neurons and glia that are associated with the modulation of locomotor activity^27,28^. The PCB-Gal4 line, described in more detail below, expresses in a sparse subset of neurons in the nervous system. The 69B and Ras2 drivers express broadly across the nervous system. As neuronal expression of the tsh enhancer trap line is biased toward the ventral nervous system (VNS)^29^, the positive result with tsh-Gal4 suggests that VNS neurons may play a role in the metabolic phenotype. To further localize the effect, we suppressed expression in tsh-Gal80+ neurons with an intersectional approach. We drove the Nf1 RNAi pan-neuronally with R57C10 in one set of flies and combined it with the tsh-Gal80 repressor to suppress expression of Nf1 RNAi in another (Fig. 2c and Supplementary Fig. 1d). The tsh-Gal80 repressor differentially removes Nf1 RNAi expression from neurons in the VNS, resulting in normal Nf1 expression levels in tsh-positive neurons^29,30^. Limiting expression with tsh-Gal80 eliminated the metabolic phenotype, suggesting that Nf1-sensitive neurons are tsh-positive, and may reside in the VNS.

The mapping experiments did not localize the Nf1 effect to major excitatory or inhibitory neurotransmitter classes. No effect was observed when knocking down Nf1 in selective sets of neurons covering major candidate neurotransmitter systems (notably GABAergic and cholinergic neurons and neurons that release insulin-like peptides). Nf1 knockdown in tyrosine decarboxylase neurons (Tdc2) did not cause an increase in metabolism despite a significant increase in CO_2_ when Nf1 is knockdown in its broadly expressed receptor (Oct-TyrR-Gal4). Instead, the driver with the most restricted expression pattern that produced a large effect was the PCB-Gal4 driver (Fig. 2d). This driver has a Gal4 enhancer trap in the pyruvate carboxylase (PCB) locus, and it has been previously termed “fatbody-Gal4” due to positive expression in the fat body^31^. However, our experiments suggest that the metabolic phenotype is neuronal in origin. Therefore, we tested whether this driver also exhibits neuronal expression. Using PCB-Gal4 to drive green fluorescent protein (mCD8::GFP), we fixed and immunostained nervous systems, and found sparse but robust GFP expression in neurons in both the brain and VNS (Fig. 2d). Given that the driver is not only fat body-specific, we refer to it by its genomic locus (PCB-Gal4).

To further examine whether the metabolic effect was due to fat body or neuronal expression, we tested three other fat body-expressing Gal4 drivers: R4, Cg, and Lsp2^32–34^. None of these produced a metabolic phenotype when knocking down Nf1 (Fig. 2a and Supplementary Fig. 2). Further, we tested two neuronal populations, which have been identified as regulating food intake and metabolism, c673a-Gal4 and fruitless-Gal4. Knockdown of Nf1 in c673a-Gal4 and two fruitless-Gal4 drivers (fru- and R23B12-Gal4) did not lead to an increase in metabolism (Fig. 2a and Supplementary Fig. 1e). Additionally, 69B-Gal4, one of the Gal4 lines with the largest effect sizes (Fig. 2a,b), has previously been shown to be expressed in the central nervous system but not in the fat body^35^. To directly test the potential role for fat body knockdown of Nf1 in metabolism via the PCB-Gal4 driver, we subtracted neuronal expression from the PCB-Gal4 expression pattern using the neuronal repressor elav-Gal80^36^ (Fig. 2e and Supplementary Fig. 3a). This eliminated the Nf1-induced metabolic effect, demonstrating that neurons in the PCB driver were responsible for the effect. Finally, subtracting neurons from the PCB-Gal4 driver with tsh-Gal80 while knocking down Nf1 eliminated the Nf1 effect on metabolism, suggesting that neurons in the VNS within the PCB driver may contribute (Fig. 2e and Supplementary Fig. 3a).

To delineate the temporal role of PCB-Gal4+ neurons in metabolism, we manipulated their activity and synaptic transmission. First, we conditionally blocked synaptic transmission with a temperature-sensitive dynamin mutant, UAS-Shibire^ts^^37^, while measuring CO_2_ production. There was no detectable change in metabolic rate when synaptic transmission was blocked (Fig. 2f and Supplementary Fig. 3b). However, acute stimulation of PCB neurons using UAS-TrpA1^38^ increased metabolic rate (Fig. 2g and Supplementary Fig. 3c). This suggests that the neuronal activity of the PCB-Gal4+ neurons dynamically modulate metabolic rate. The lack of effect with shibire^ts^ blockade suggests one of three possibilities: the neurons may have a low basal firing frequency in the normal fed state, partially redundant circuits could regulate metabolic rate, or the shibire^ts^ blockade could be incomplete. Overall, these experiments add additional evidence that neurons in the PCB-Gal4 driver are responsible for the metabolic phenotype, and suggest that their activity dynamically regulates metabolism.

**Fig. 3:**
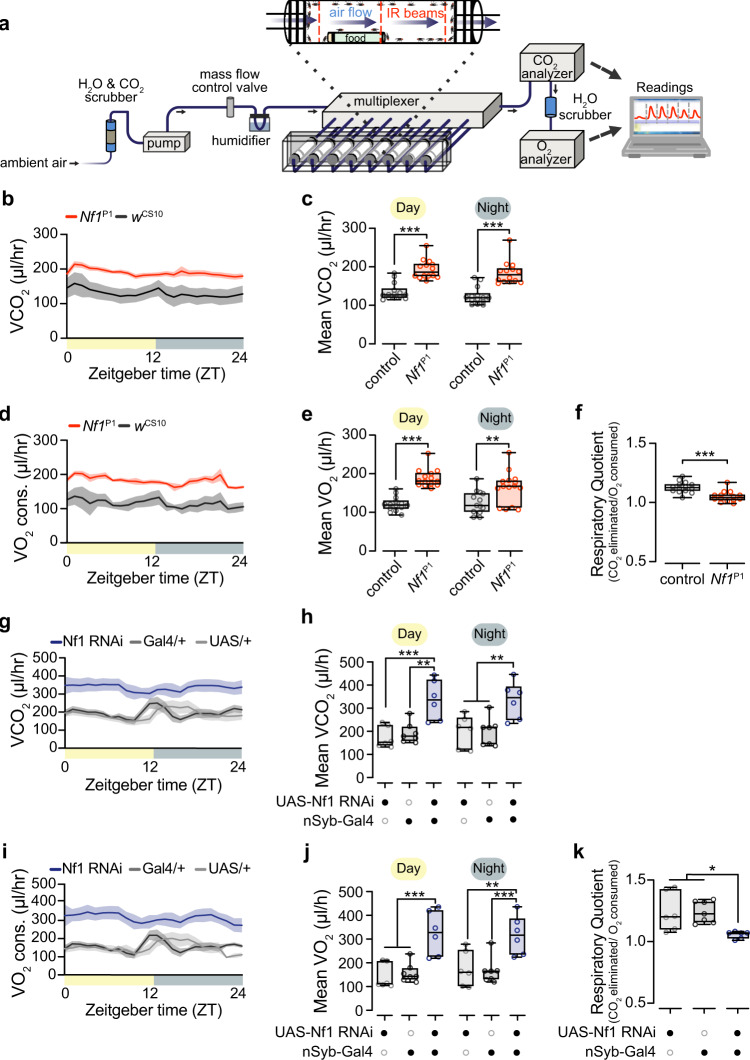
Loss of Nf1 increases metabolic rate across the circadian photoperiod. **a** O_2_ consumption and CO_2_ production, measured with indirect calorimetry. **b** CO_2_ production measured in *Nf1*^P1^ mutants and *w*^CS10^ controls, plotted in 1-h bins across the circadian photoperiod. **c** Quantification of CO_2_ production from (**b**) during day and night periods. ***p < 0.001 (Sidak, two-sided; *n* = 14 per genotype). **d** O_2_ consumption measured in *Nf1*^P1^ mutants and *w*^CS10^ controls. **e** Quantification of O_2_ consumption from (**d**) during day and night periods. Day experimental ***p < 0.001 re: control, night experimental **p = 0.003 re: control. (Sidak, two-sided; day: *n* = 14 per genotype, night: *n* = 13 control, *n* = 14 *Nf1*^P1^). **f** Respirometry quotient in *Nf1*^P1^ mutants and *w*^CS10^ controls. ***p < 0.001 (Mann–Whitney, two-sided; *n* = 14). **g** CO_2_ production, comparing pan-neuronal nSyb > RNAi with heterozygous controls. **h** Quantification of CO_2_ production from (**g**) during day and night periods. Day experimental ***p = 0.001 re: UAS-Nf1 RNAi/+, **p = 0.001 re: nSyb-Gal4/+. Night experimental **p = 0.004 re: UAS-Nf1 RNAi/+, **p = 0.002 re: nSyb-Gal4/+. (Sidak, two-sided; *n* = 6 UAS-Nf1 RNAi/+, *n* = 7 nSyb-Gal4/+, *n* = 6 experimental group). **i** O_2_ consumption, comparing pan-neuronal nSyb > RNAi lines with heterozygous controls. **j** Quantification of O_2_ production from (**i**) during day and night periods. Day experimental ***p = 0.0001 re: UAS-Nf1 RNAi/+, ***p = 0.003 re: nSyb-Gal4/+. Night experimental **p = 0.003 re: UAS-Nf1 RNAi/+, ***p = 0.001 re: nSyb-Gal4/+. (Sidak, two-sided; *n* = 6 UAS-Nf1 RNAi/+, *n* = 7 nSyb-Gal4/+, *n* = 6 experimental group). **k** Respirometry quotient in nSyb > RNAi lines and heterozygous controls. Experimental *p = 0.014 re: UAS-Nf1 RNAi, *p = 0.011 re: nSyb-Gal4/+ (Sidak, two-sided; *n* = 6 UAS-Nf1 RNAi/+, *n* = 7 nSyb-Gal4/+, *n* = 6 experimental group). Each “*n*” represents a single respirometer containing 25 animals. Box plots—box: 1st to 3rd quartiles, median: line, whiskers: min–max, individual data points: circles. Data are presented as mean values ± SEM in (**b**, **d**, **g**, and **i**).

To gain further insight into the temporal parameters and mechanistic underpinnings of the metabolic phenotype, we turned to stop-flow respirometry, quantifying O_2_ consumed and CO_2_ produced (Fig. 3a)^39^. Mutant *Nf1*^P1^ adult flies exhibited increased CO_2_ production and O_2_ consumption across the 24-h photoperiod relative to controls (Fig. 3b, d). The total daytime and nighttime metabolic rate was elevated in *Nf1*^P1^ mutants compared to controls (Fig. 3c, e). Similarly, when Nf1 was knocked down pan-neuronally using nSyb-Gal4, we observed increased O_2_ consumption and CO_2_ production across the circadian photoperiod relative to controls (Fig. 3g–j). In both cases, the respiratory quotient (RQ), the ratio of CO_2_ eliminated to O_2_ consumed, was significantly reduced (Fig. 3f, k). Decreased RQ is consistent with increased utilization of endogenous fat stores^39^, suggesting that loss of Nf1 may increase fat utilization, a possibility we consider further below. Overall, these data provide independent support for the role of Nf1 in metabolic regulation, demonstrate that it is consistent across the 24-h photoperiod, and suggest that it may result from altered fat homeostasis.

### Metabolic regulation is independent of grooming

**Fig. 4:**
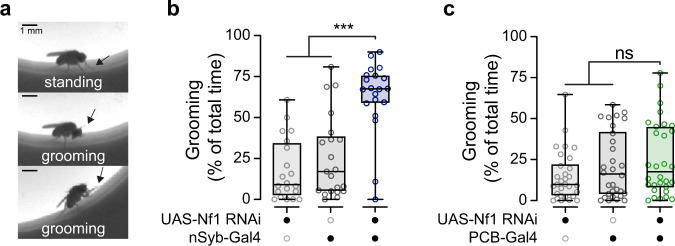
Knockdown of Nf1 in PCB-Gal4+ neurons does not affect spontaneous grooming. **a** Representative images of fly grooming in an open field. Top panel: a fly standing in place (not grooming). Middle panel: a fly grooming its head. Bottom panel: a fly grooming its prothoracic (front) legs. In all panels, an arrow points to the prothoracic leg location. Scale bars = 1 mm. **b** Quantification of grooming in flies in which Nf1 has been knocked down pan-neuronally with the nSyb-Gal4 driver. ***p < 0.001 (Kruskal–Wallis, two-sided; *n* = 20 UAS-Nf1 RNAi/+, *n* = 21 nSyb-Gal4/+, *n* = 21 experimental group). **c** Quantification of grooming in flies in which Nf1 has been knocked down with the PCB-Gal4 driver. ns: not significant. *p* = 0.209 (Kruskal–Wallis, two-sided; *n* = 30). Each “*n*” = single fly. Box plots—box: 1st to 3rd quartiles, median: line, whiskers: min–max, individual data points: circles. ns: not significant.

Loss of Nf1 increases spontaneous grooming^22^, which could drive an increase in energy expenditure. To test whether this accounts for the increase in metabolic rate observed here, we knocked down Nf1 and quantified grooming in an open field arena (Fig. 4a). Pan-neuronal knockdown of Nf1 elevated spontaneous grooming (Fig. 4b), as previously reported^22^. In addition, we tested the more restricted PCB-Gal4 driver. While knocking down Nf1 with this driver produced one of the largest increases in metabolic rate (Fig. 2a, b), it did not significantly elevate spontaneous grooming (Fig. 4c). Therefore, Nf1 functions in independent populations of neurons to regulate metabolic rate and grooming, and the elevated metabolic rate observed in *Nf1* mutant flies is not due to changes in grooming activity.

### Altered starvation susceptibility and lipid turnover kinetics

**Fig. 5:**
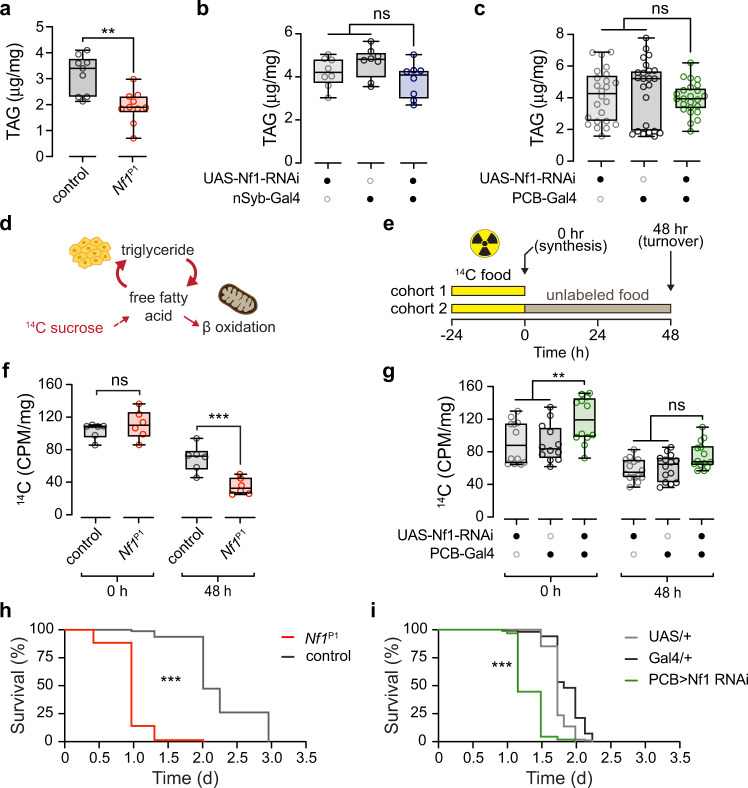
Loss of Nf1 reduces starvation resistance and alters lipid stores and turnover kinetics. **a** Quantification of total body triglyceride (TAG) content in *Nf1*^P1^ mutants (*n* = 9) and *w*^CS10^ controls (*n* = 10). **p = 0.002 (Mann–Whitney, two-sided). **b** Quantification of total TAG content of pan-neuronal nSyb-Gal4 > Nf1 RNAi compared to heterozygous controls. *p* = 0.095 (One-way ANOVA, two-sided; *n* = 8). **c** Quantification of total TAG content of PCB-Gal4 > Nf1 RNAi compared to heterozygous controls. *p* = 0.552 (Kruskal–Wallis, two-sided; *n* = 24). **d** Diagram of ^14^C sucrose radiolabeling and lipid turnover. **e** Experimental protocol of ^14^C sucrose radiolabeling for lipid turnover analysis. **f** Quantification of lipid turnover of *Nf1*^P1^ mutants via ^14^C sucrose radiolabeling. Radioactivity was measured as counts per minute (CPM) and normalized to fly weight (mg). At time point zero hours (0 h) *p* = 0.604, at 48-hours (48 h) ***p < 0.001, (Sidak, two-sided; *n* = 6 for both genotypes at both time points). **g** Quantification of lipid turnover of PCB-Gal4 > Nf1 RNAi via ^14^C sucrose radiolabeling. Radioactivity was measured as counts per minute (CPM) and normalized to fly weight (mg). At time point zero hours (0 h) experimental **p = 0.010 re: UAS-Nf1 RNAi/+, **p = 0.006 re: PCB-Gal4/+. At 48-hours (48 h) experimental *p* = 0.129 re: UAS-Nf1 RNAi, *p* = 0.252 re: PCB-Gal4/+. (Sidak, two-sided; *n* = 12 at 0-h, *n* = 14 at 48-h). **h** Lifespan under starvation, comparing *Nf1*^P1^ mutants (*n* = 78) and *w*^CS10^ controls (*n* = 80). ***p < 0.001 (*X*2(1) = 154.0, Mantel–Cox test, two-sided). **i** Lifespan under starvation, comparing PCB-Gal4 Nf1 knockdown (PCB > Nf1 RNAi) (*n* = 119) and UAS/+ (*n* = 120) and Gal4/+ (*n* = 100) controls. ***p < 0.001 (*X*2(2) = 281.3, Mantel–Cox test, two-sided). Each “n” in (**a**–**c**) represents a sample containing five animals. Each “*n*” in (**f**, **g**) represents a sample containing twenty animals. Box plots—box: 1st to 3rd quartiles, median: line, whiskers: min–max, individual data points: circles. ns: not significant.

Alterations in metabolic rate could affect lipid storage, and the decreased respirometry quotient suggests that lipid stores may be reduced (along with increased lipid utilization). To directly test this, we first quantified triglyceride content using coupled colorimetry^40^. Triglyceride stores were significantly reduced in *Nf1*^P1^ mutants compared to controls (Fig. 5a). When knocking down Nf1 with RNAi, either pan-neuronally via nSyb-Gal4 or in PCB-Gal4+ neurons, there were no significant differences in whole-body triglyceride levels (Fig. 5b, c). Changes in either lipogenesis or lipolysis can occur regardless of whether triglyceride levels are affected (Fig. 5d). To determine whether the kinetics of lipid turnover were altered, we examined lipid turnover in a pulse-chase experiment (Fig. 5e). Flies were fed radiolabeled ^14^C sucrose for 24 h, and incorporation of ^14^C sucrose into fatty acids in the flies was measured with scintillation at 0 h or 48 h after returning flies to normal, unlabeled food. Comparing *Nf1*^P1^ mutants with controls, the initial incorporation of ^14^C did not differ (Fig. 5f). However, 48 h after returning flies to unlabeled food, *Nf1*^P1^ flies retained significantly less ^14^C than controls. This suggests that *Nf1*^P1^ mutants exhibited significantly elevated rates of lipolysis, which resulted in faster clearing of ^14^C following return to unlabeled food. We next tested the effect of knocking down Nf1 in PCB-Gal4+ neurons via RNAi, using the same ^14^C radiolabeling protocol. In this case, we observed a significant increase in ^14^C scintillation counts following 24 hr on radiolabeled food in the knockdown group, with no significant difference after returning to unlabeled food for 48 h (Fig. 5g). This suggests an increase in lipid turnover rate in the Nf1 knockdown group, with an increase in the initial rate of ^14^C incorporation and a slightly faster loss of ^14^C following the return to unlabeled food. The effect was smaller than the *Nf1*^P1^ mutant, suggesting that the neurons in the PCB-Gal4 driver encompass part of the circuit/mechanism responsible for the effect. These results were significant regardless of whether incorporation was normalized to body weight or the number of flies (Supplementary Fig. 4). Together, these findings suggest that energy stores and turnover kinetics are altered in Nf1 loss of function.

To determine whether the alterations in energy stores and increased metabolic rate impact survival in the absence of food, we measured the starvation resistance of *Nf1*^P1^ mutants and RNAi lines. *Nf1*^P1^ mutants were significantly more susceptible to starvation than controls, succumbing in less than half the time of control flies when housed on a nutrient-free agar media (Fig. 5h). Likewise, Nf1 knockdown in PCB-Gal4+ neurons increased starvation susceptibility (Fig. 5i). These results suggest that loss of Nf1 increases starvation susceptibility through increased lipid turnover, which depletes energy stores more quickly when the animals are without food. Further, these data suggest that neurons in the PCB-Gal4 expression pattern contribute to a portion of this effect.

### Loss of Nf1 increases feeding in adult flies

**Fig. 6:**
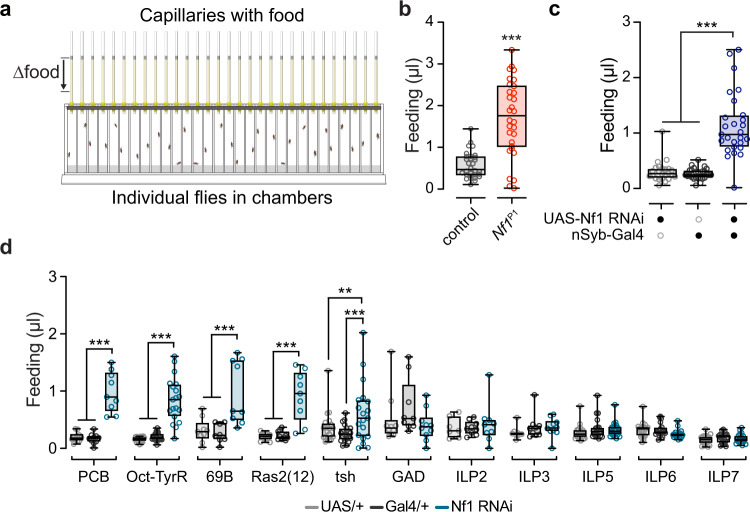
Loss of Nf1 increases feeding. **a** Illustration of the capillary feeding apparatus. **b** Feeding in *Nf1*^P1^ mutants and *w*^CS10^ controls. ***p < 0.001 (Mann–Whitney, two-sided; *n* = 30 per genotype). **c** Feeding with pan-neuronal Nf1 RNAi (driven by nSyb-Gal4) and heterozygous Gal4/+ and UAS/+ controls. ***p < 0.001 (Dunn’s test, two-sided; *n* = 20 per genotype). **d** Feeding measured with knockdown of Nf1 in different subsets of neurons with various Gal4 drivers. *p < 0.05, **p < 0.01, ***p < 0.001 (Sidak, two-sided; *n* = 7–20). Exact sample sizes and *p*-values found in Source data file for (**d**). Each “*n*” represents a single fly in a chamber. Box plots—box: 1st to 3rd quartiles, median: line, whiskers: min–max, individual data points: circles.

Feeding and energy stores are homeostatically regulated, and animals often compensate for depletion of energy stores by increasing food intake. Since the loss of Nf1 increased metabolic rate, altered lipid stores and lipid kinetics, energy intake could be increased as a homeostatic compensatory mechanism. To test this, we examined feeding using a capillary feeding assay^41^ (Fig. 6a). Food intake was significantly increased in *Nf1*^P1^ adult flies relative to controls (Fig. 6b). Knocking down Nf1 pan-neuronally also increased feeding relative to controls (Fig. 6c), confirming the effect and suggesting that it was due to the loss of Nf1 specifically in neurons. We next tested the neuronal circuit requirements for Nf1 in the context of feeding. A selection of Gal4 drivers that labeled different neurochemical subsets, and/or produced metabolic alterations, were used to drive Nf1 RNAi (Fig. 6d). Among these lines, significant elevations in feeding were observed when Nf1 was knocked down pan-neuronally (nSyb) (Fig. 6c), biased toward the VNS (tsh), and in subsets of neurons labeled by the 69B, Ras2(12), Oct-TyrR, and PCB-Gal4 drivers (Fig. 6d). No effect was observed when Nf1 was knocked down in GABAergic neurons (GAD) or insulin-like peptides (ILP2, ILP3, ILP5, ILP 6, ILP7) (Fig. 6d). Thus, knocking down Nf1 in the same circuits that elevated metabolic rate (Fig. 2a, b) also drove an increase in feeding. While it is possible that Nf1 independently regulates both phenotypes via actions in the same circuit(s), we surmise that the increase in feeding is likely a homeostatic response to the increase in metabolic rate and alterations in lipid homeostasis caused by loss of Nf1.

### Ras GRD signaling underlies the Nf1 metabolic alterations

**Fig. 7:**
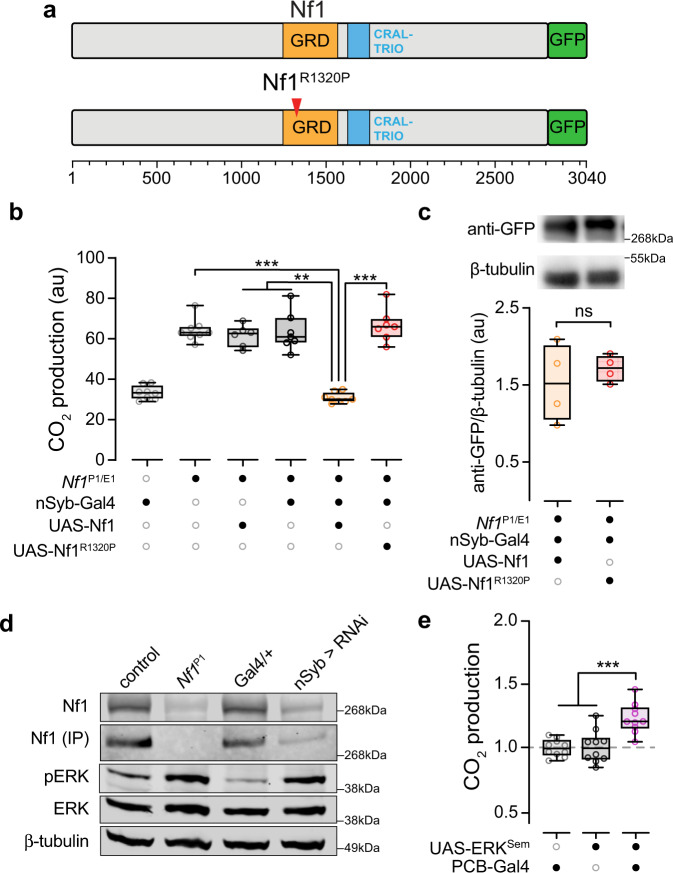
Nf1 metabolic effects are rescued by full-length Nf1 and require a functional GAP-related domain (GRD). **a** Major domains in neurofibromin (GRD and CRAL-TRIO), site of the R1320P mutation (red arrow), and GFP C-terminal fusion. **b** Transgenic expression of full-length Nf1 and Nf1^R1320P^ in the heteroallelic *Nf1*^P1/E1^ mutant background. Full length Nf1 rescues CO_2_ production to nSyb-Gal4/+ levels. Experimental (UAS-Nf1; nSyb-Gal4, *Nf1*^P1/E1^) ***p = 0.001 re: *Nf1*^P1/E1^, **p = 0.009 re: UAS-Nf1; *Nf1*^P1/E1^, **p = 0.005 re: nSyb-Gal4, *Nf1*^P1/E1^, ***p = 0.0003 re: UAS-Nf1^R1320P^; nSyb-Gal4, *Nf1*^P1/E1^. (Dunn’s test, two-sided; *n* = 8 nSyb-Gal4/+, *n* = 8 *Nf1*^P1/E1^, *n* = 6 UAS-Nf1; *Nf1*^P1/E1^, *n* = 7 nSyb-Gal4, *Nf1*^P1/E1^, *n* = 8 UAS-Nf1; nSyb-Gal4, *Nf1*^P1/E1^, *n* = 7 UAS-Nf1^R1320P^; nSyb-Gal4, *Nf1*^P1/E1^). **c** Representative western blot of anti-GFP and β-tubulin shown. Samples derived from the same experiment and processed in parallel. Blot normalized to the intensity of loading control (β-tubulin). Quantification represents four replicates. au: arbitrary units, ns: not significant. *p* = 0.510 (Student’s *t*-test, two-sided; *n* = 4 per genotype). **d** Representative western blot of Nf1 and pERK levels in *Nf1*^P1^ and Nf1 RNAi flies compared to genetic controls (*w*^CS10^ and Gal4/+, respectively). Samples derived from the same experiment and processed in parallel, with β-tubulin as loading control on the same blots. Representative blot shown from one of two independent experiments. **e** Normalized CO_2_ production with expression of an ERK gain-of-function mutation (UAS-ERK^Sem^) in PCB-Gal4. ***p < 0.001 (Sidak, two-sided; *n* = 10 per genotype). Each “*n*” = in (**b**, **e**) represents a single respirometer (containing 4 animals). Box plots—box: 1st to 3rd quartiles, median: line, whiskers: min–max, individual data points: circles.

Catalytic Ras GAP activity of neurofibromin is mediated by its central GRD^3,4^. To determine whether Ras signaling is critical for mediating Nf1 effects on metabolic rate, we tested the role of the Nf1 GRD. The Gal4/UAS system was used to express Nf1 rescue constructs in flies with the heteroallelic *Nf1*^P1/E1^ mutant combination. Pan-neuronal rescue of full-length, wild-type Nf1 restored metabolic rate to control levels (Fig. 7a, b). In contrast, pan-neuronal expression of full-length Nf1 carrying a missense mutation in the “arginine finger” of the GRD, R1320P, did not rescue the metabolic effect (Fig. 7a, b). This missense mutation corresponds to the patient-derived R1276P mutation in *NF1* shown to reduce Ras GAP activity >1000 fold^16,42^. Western blot analysis showed no significant changes in protein expression between pan-neuronal rescue of full-length, wild-type Nf1 and pan-neuronal expression of full-length Nf1 carrying the R1320P missense mutation (Fig. 7c), suggesting that the mutation did not affect protein stability. These data suggest that the expression of wild-type Nf1 in neurons restores normal metabolic rate and that RasGAP activity is necessary for Nf1 metabolic effects. Additionally, the Nf1^R1320P^ mutation does not affect the stability/degradation of the Nf1 protein.

Nf1 protein levels and downstream signaling were examined with western blot and immunoprecipitation (IP) in RNAi lines and *Nf1*^P1^ mutants. We confirmed that Nf1 was eliminated in *Nf1*^P1^ mutants and reduced with RNAi knockdown (Fig. 7d). To examine the effect of Nf1 loss-of-function on downstream canonical MAP kinase signaling, we also examined phosphorylated ERK (pERK) and total ERK. In both *Nf1*^P1^ mutants and Nf1 RNAi lines, there was an increase in pERK, with no detectable change in total ERK levels (Fig. 7d). These results confirm that both loss-of-function conditions elevate pERK levels. To test whether ERK activation is a major driver of the metabolic phenotype, we expressed an ERK transgene containing a mutation rendering it constitutively active, UAS-ERK^Sem^. Activation of ERK in PCB-Gal4+ neurons increased CO_2_ production, as assayed by respirometry (Fig. 7e), partially phenocopying the effect of knocking down Nf1 in these neurons (Fig. 2e). This suggests that activation of ERK contributes to the enhancement of metabolic rate and increase in CO_2_ production following loss of Nf1.

## Discussion

This study provides mechanistic insight into the role of Nf1 in regulating basal metabolic rate via neuronal mechanisms in *Drosophila*. The data support the following major specific conclusions about the role of Nf1 in regulating metabolic rate: (1) loss of Nf1 increases metabolic rate, independent of locomotor activity (grooming), (2) loss of Nf1 function alters energy stores and turnover kinetics, while increasing starvation susceptibility and food consumption, (3) Nf1 modulates metabolism via a sparse subset of neurons labeled by the PCB-Gal4 driver, and (4) Nf1 acts via the GRD and downstream ERK signaling to alter metabolic function.

We found that Nf1 plays a central role in regulating metabolic rate via both indirect and stop-flow respirometry. Both genomic mutations and pan-neuronal knockdown of Nf1 significantly elevated metabolic rate. We tested multiple driver lines to determine which cell types are most affected by Nf1 loss of function, finding that Nf1 acts on a relatively narrow set of neurons to modulate metabolic rate. These neurons were not ones commonly implicated in metabolism, feeding, or organismal growth, as knocking down Nf1 in insulin-producing cells, peptidergic neurons, ring gland cells, monoaminergic neurons, obesity-blocking neurons, etc., produced no effect. It was particularly surprising that loss of Nf1 function in the insulin-producing cells yielded no significant changes in metabolism or feeding, as the insulin-like peptides released by these neurons are functional homologs of insulin and insulin-like growth factors. Furthermore, alterations to such cells affect starvation response and lipid stores^43^. Overall, our study revealed a central modulatory effect of Nf1 on metabolic rate, though Nf1 could exert metabolic effects via actions on peripheral tissues as well.

A sparse set of neurons in the nervous system, labeled most discretely by the PCB-Gal4 driver, was responsible for the Nf1 effect on metabolism. Acute activation of PCB-Gal4 neurons increased metabolic rate significantly, while blocking these neurons did not affect metabolism. These results suggest that loss of Nf1 may hyperactivate these neurons, leading to metabolic changes. Additionally, the baseline firing rate of PCB neurons is likely low, given that inhibiting their synaptic release did not affect metabolic rate. Due to additional fat body expression with this driver, we verified that the metabolic phenotype was due to loss of neuronal Nf1, using additional Gal4 lines that express in the adult and larval fat body. The fat body serves an important homeostatic function by storing lipids, similarly to white adipose tissue in mammals, and plays a role in regulating peripheral tissues under both fed and starved conditions^43^. Therefore, the lack of metabolic effects with multiple fat body-specific drivers was notable. The effects of Nf1 on metabolic rate could theoretically be due to altered energy expenditure within neurons themselves or central control of metabolism. Our experiments implicate the latter, as the PCB-Gal4 driver labels a sparse neuronal population and drives a robust metabolic effect when used to knock down Nf1. Additional drivers produced similar phenotypes when knocking down Nf1, ranging from pan-neuronal drivers to the Oct-TyrR-Gal4 driver. Given the far broader expression of these drivers^23^, it is likely that the PCB-Gal4 labels a subset of neurons within the broader driver expression patterns.

We propose that the loss of Nf1 in PCB-Gal4+ neurons drives a series of metabolic effects which trigger a homeostatic increase in feeding. Most of the metabolic phenotypes (increased metabolic rate, altered lipid turnover, and increased starvation susceptibility) depend on PCB-Gal4+ neurons, and it is unlikely that the motor circuits regulating feeding would be the same as the circuits regulating all of the others. Some broader neuronal drivers (69B, ChAT, Ras2) also produced metabolic effects. These drivers may overlap with the PCB-Gal4+ subset. One broad driver that elevated metabolic rate was Oct-TyrR-Gal4, suggesting that the neurons modulating metabolic rate may express the Oct-TyrR receptor. This receptor responds to both octopamine and tyramine^44^, suggesting the potential neurotransmitter complement of candidate upstream neurons. Previous studies from our group have described increased grooming activity in *Nf1*^P1^ mutants and with pan-neuronal RNAi^22,23^. Increased locomotor activity, including activity such as grooming, might be expected to drive increases in metabolism. However, the Nf1 metabolic and grooming phenotypes were dissociable with the PCB-Gal4 driver, which increased metabolic rate without increasing grooming. Thus, we surmise that the effects of Nf1 on grooming and metabolic rate are caused by distinct neuronal circuits, with little, if any, overlap. A thorough analysis of the neuronal circuit driving both phenotypes (particularly grooming) will be necessary to understand how Nf1 independently modulates these behaviors.

The metabolic effects we observed in the *Drosophila* model, combined with the conserved signaling and cellular functionality, suggest that loss of Nf1 could produce metabolic alterations across taxa. Ras is upstream of multiple signaling cascades that influence cellular metabolism, including mTOR and ERK. mTOR and ERK modulate gluconeogenesis, protein synthesis, adipocyte differentiation, lipid/cholesterol homeostasis, lipogenesis, and lipolysis^5^. Conditional knockout of Nf1 in mouse models, while rapidly lethal, produces metabolic alterations in muscle, including increased triglyceride content and activities of oxidative metabolism enzymes^45^. Further, mouse embryonic fibroblasts lacking Nf1 show changes in basal metabolic rate and mitochondrial bioenergetics^13^. These findings, along with data from the present study, suggest the potential for homeostatic regulation of metabolic rate via Nf1-mediated mechanisms. Our data further support a role for ERK, in particular, in driving Nf1 effects on metabolism. This is consistent with studies documenting Nf1/ERK-mediated modulation of mitochondria in an oncogenic context^13^, as well as the rescue of brain abnormalities in a mouse model using ERK inhibitor treatment^46^. Similarly, a current treatment strategy for plexiform neurofibromas in NF1 involves MEK inhibitors^47^.

The most well-characterized biochemical function of neurofibromin is its RasGAP activity mediated by the GRD. In the present experiments, transgenic expression of full-length, wild-type neurofibromin restored normal metabolic rate in *Nf1* mutants. Expression of an Nf1 transgene containing the catalytically dead R1320P mutation failed to rescue. This mutation recapitulates the human patient-derived R1276P mutation that impacts the arginine finger of the GRD, reducing RasGAP activity >1000 fold^42^. In addition to the GRD, neurofibromin contains other domains with potential functional significance, such as a lipid-binding CRAL-TRIO domain (Fig. 7a). Western blot analysis showed no difference between protein levels of the R1320P mutation and full-length Nf1, suggesting that Nf1 protein stability is not affected by the R1320P mutation. Thus, this experiment suggests that catalytic activity from the GRD was critical for Nf1 dependent modulation of metabolic rate. Previous studies have implicated Ras/GRD function in Nf1 phenotypes^16,17,48^, and some have identified signaling roles involving dopaminergic signaling, G protein signal transduction, and cAMP signaling (some of which are activated downstream of Ras)^6,14,15,49–53^. The effects of aberrant Ras signaling could include transcriptional regulation, as Ras regulates the activity of multiple transcription factors^13^. Further, alterations in these signaling cascades may affect the release of neurotransmitters in ways that produce non-cell-autonomous effects on signaling cascades in other cells^16^. It is important to note that our data suggest that the functional GRD is necessary for Nf1-dependent modulation of metabolic rate, but do not exclude other potential molecular routes for Nf1 effects.

Among individuals with NF1, there are subtle alterations in growth, particularly reduced stature^54^, as well as reduced body mass index in males^8^. Alterations in specific metabolites have been reported, though these are sex-specific^8^. Diabetes mellitus and deaths from noted diabetes complications are rare in patients with NF1^8^. Loss of NF1 increases glycolysis and decreases respiration via mitochondrial ERK signaling, which may play a role in tumor growth^13^. Finally, increased resting energy expenditure has been reported in a cohort of female NF1 patients as well as a decrease in respiratory quotient compared to healthy controls^11^. In mice, Nf1 heterozygosity reduces fat mass and alters various aspects of metabolism, such as glucose utilization, reminiscent of human NF1^12^. Nf1 knockdown also decreases glycogen in *Drosophila*^55^, along with the phenotypes reported here. Therefore, loss of Nf1 has the potential to regulate cellular and organismal metabolism, and these alterations in metabolism may contribute to the pathophysiology of NF1. Our data suggest that changes in neuronal metabolic control may be a feature of the cellular and organismal alterations that occur following the loss of Nf1.

## Methods

### *Drosophila* husbandry and stocks

Flies were cultured on cornmeal/agar food medium according to standard protocol and housed at 25 °C, 60% relative humidity, on a 12:12 light:dark cycle. The *Nf1*^P1^ mutation was backcrossed six generations into the *w*^CS10^ genetic background and *w*^CS10^ flies were used as controls. Genetic control for *Nf1*^P1/E1^ trans-heterozygotes is *w*^CS10^/w^iso2;3^. UAS-Nf1-eGFP transgenes were generated using an *Nf1* mini-gene (full-length *Nf1* cDNA corresponding to the RF isoform with addition of introns 9 and 10). The R1320P mutation was created in a wild-type cDNA using the Q5 Site-Directed Mutagenesis Kit (New England Biolabs). Wild-type and R1320P mutant *Nf1* were then subcloned into the pUAST-attB vector with an in-frame C-terminal fusion with eGFP cDNA. Transgenic lines were produced by integrating the constructs at the attp40 site (Rainbow Transgenic Flies Inc.) The Nf1 RNAi line was obtained from the Vienna *Drosophila* RNAi Center (VDRC #109637), Gal4/+ control crosses consisted of an empty attP control line (VDRC #60100). UAS-dicer2 was included to potentiate the RNAi effect^56^, and was included with the UAS-RNAi (i.e., all experimental and UAS/+ genotypes). ERK was activated via expression of ERK with the gain-of-function mutation ERK^Sem^ (UAS-rl^Sem^)^57^. The following lines were obtained from the Bloomington *Drosophila* Stock Center (BDSC): nSyb (BDSC #51635), R57C10 (39171), 69B (1774), tsh-Gal4 (3040), and Oct-TyrR-Gal4 (36494). PCB-Gal4 (fatbody-Gal4) was previously generated by Ronald Kühnlein, and Ras2 insertions by James Walker. Gal4 lines used in the screen were obtained from the BDSC or public sources. Male flies were used for all experiments to prevent egg accumulation in behavioral and respirometry chambers.

### CO_2_ measurement using respirometry

CO_2_ production was measured with respirometry^24^. Briefly, a 1 ml pipette tip and 50 µl capillary micropipette were securely glued together. Soda lime was placed into each pipette tip between two foam pieces to avoid contact with flies. Sixteen respirometers were hung on a custom-made rack in a latch-lid measurement chamber. Flies were anesthetized with CO_2_ and allowed to recover for at least 24 h before beginning the experiment. Four flies of the same genotype were placed into each pipette using an aspirator and tightly sealed at the top using non-hardening modeling clay. Pipettes that were not tightly sealed were not included in final measurements. One pipette was left empty in each chamber as a temperature and atmospheric control. The measuring chamber was filled with a red-dye solution made with a water-based dye. The latch-lid chamber with flies were left to equilibrate to incubator conditions (25 °C) for 1 h before starting the experiment (unless otherwise stated). Vacuum grease was applied between the latch-lid and chamber to reduce atmospheric and temperature fluctuations. Images were captured every 15 min using a time-lapse image software (PhenoCapture 3.3) and analyzed using Fiji 2.0, measuring the liquid meniscus position in each capillary. This measurement was subtracted from a reference image and repeated at each time point for 3 h. Data were normalized by calculating the mean CO_2_ production of control genotypes, and normalizing each data point to that value.

### Indirect calorimetry

Metabolic rate and activity were measured using the Sleep and Activity Metabolic Monitor (SAMM) system (Sable Systems International). Group-housed adult flies were measured at 25 °C, through indirect calorimetry using a stop-flow, push-through respirometry system^39,58^. Briefly, the experimental system assessed baseline CO_2_ levels from an empty chamber to measure CO_2_ production and O_2_ consumption from 25 male adult flies. Air was flushed from each chamber for 50 sec to provide a readout of CO_2_ accumulation and O_2_ consumption over a 10-min period. A H_2_O and CO_2_ scrubber was used to dehumidify and remove CO_2_ from air before being pumped into a mass flow control valve to maintain a consistent flow rate. The air was then re-humidified by passing through a reservoir containing deionized water prior to reaching the behavioral chambers. Each behavioral chamber (70 mm long × 20 mm diameter glass tube) contained 25 adult male flies with a food vial containing 1% agar and 5% sucrose. Flies were allowed to acclimate to the system for 24 h before the start of each experiment. All experimental runs included a food-only control baseline chamber. CO_2_ production was analyzed using a LI-7000 CO_2_/H_2_O Analyzer (LI-COR) and then dehumidified using an H_2_O scrubber before O_2_ consumption was measured Oxilla Dual Absolute Differential Oxygen Analyzer (LI-COR). CO_2_ production and O_2_ consumption recordings were taken every 10 min. Respirometry quotient (RQ) was calculated as the volume of CO_2_ eliminated/O_2_ consumed.

### Western blot analysis

For assessing expression of Nf1 and levels of pERK/ERK (Fig. 1e), lysates of heads from 2 to 3-day old adult flies were prepared using IP buffer (50 mM Tris-HCl pH 7.5, 125 mM NaCl, 1.5 mM MgCl_2_, 0.2% NP-40, 5% glycerol) supplemented with protease inhibitors (Complete Protease Inhibitor Cocktail, Sigma) and phosphatase inhibitors (NaF/Na_3_VO_4_/β-glycerophosphate). Immunoprecipitations were performed using 400 µg of total protein in 1 ml IP buffer incubated with 200 μl of mAb21 anti-Nf1 monoclonal antibody supernatant^14^ for 1 h at 4 °C. Immunocomplexes were precipitated with a mix of Protein A- and Protein G-Sepharose beads (1:1, Sigma) for 1 h at 4 °C before washing three times with IP buffer and denaturing with 2× SDS sample buffer. Lysates and immunoprecipitates were resolved on NuPAGE 3–8% Tris-Acetate (for Nf1) or NuPAGE 4–12% Bis-Tris gels (pERK/ERK/β-tubulin). After transfer to nitrocellulose membranes, blots were processed according to the Odyssey CLx protocol (LI-COR). Antibodies used for immunoblotting were: anti-Nf1 (mouse, mAb21, 1:10), anti-phospho-ERK (mouse, Sigma M8159, 1:2500), total ERK (rabbit, CST 9102, 1:1000), anti-β-tubulin (mouse, Developmental Studies Hybridoma Bank E7, 1:10,000), anti-mouse IRDye secondary antibody (goat, Invitrogen, A32730, 1:10,000), and anti-rabbit IRDye (donkey, Invitrogen, A10043, 1:10,000) secondary antibody.

For assessing expression of Nf1-eGFP transgenes (Fig. 7c), lysates of 5-day old adult fly heads were prepared using RIPA buffer supplemented with HALT protease and phosphatase inhibitors (Thermofisher Scientific). Protein samples were mixed with NuPAGE 4× LDS Sample Buffer (Invitrogen), resolved on NuPAGE 3–8% Tris-Acetate Protein Gels with NuPAGE Tris-Acetate SDS Running Buffer supplemented with NuPAGE Antioxidant. SDS-PAGE gels were transferred to polyvinylidene difluoride (PVDF) membranes and 5% BSA in TBST was used for blocking. Antibodies used were: rabbit anti-GFP (Invitrogen, A11122, 1:4000), and mouse anti-β-tubulin (DHSB, E7, 1:2000). The membranes were washed with TBST, followed by incubation for 1 h at room temperature after the addition of horseradish peroxidase-conjugated anti-Rabbit IgG (H + L) (Jackson ImmunoResearch, 711035152, 1:5000) and horseradish peroxidase-conjugated anti-Mouse IgG (H + L) (Jackson ImmunoResearch, 715035151, 1:5000). After washing, WesternBright Quantum HRP substrate (Advansta) was added to the membranes and images were captured with AlphaImager HP system (ProteinSimple), and analyzed using Fiji 2.0. Images of blots found in the Source Data file.

### Immunohistochemistry

Five to seven-day-old PCB-Gal4 adult nervous systems were dissected in 1% paraformaldehyde in S2 medium and processed as previously described^59^. Samples were stained with primary antibodies for 3 h at room temperature and at 4 °C overnight, followed by secondary antibodies for 3 h at room temperature and 4 d at 4 °C. Incubations were performed in blocking serum (3% normal goat serum). Samples were mounted in Vectashield (Vector Laboratories) for analysis. The following antibodies were used: rabbit anti-GFP (1:1000, Invitrogen), mouse anti-nc82 (1:50, DSHB), goat anti-rabbit IgG, and goat anti-mouse IgG (1:800, Alexa 488 or Alexa 633, respectively, Invitrogen). Leica TCS SP8 confocal microscope with LAS X software was used to obtain images.

### Total grooming

Grooming was quantified with behavioral analysis in an open field^22^. Individual animals were aspirated into an open field area, habituated for 15 min, and a 5-min video recording was recorded for analysis. Videos were collected using the Image Acquisition Tool in Matlab. Grooming was manually scored by watching videos frame-by-frame in Matlab R2015b and start and stop frames were recorded for each grooming event. Grooming was calculated as the percent of total time grooming during the 5-min video.

### Coupled colorimetric assay for TAG

Triglyceride content was quantified with a coupled colorimetric assay^40^. Samples were rinsed in PBS, rapidly homogenized in PBST, and the supernatant was heated at 70 °C for 10 min. The glycerol standard solution (Sigma 2.5 mg/ml triolein equivalent glycerol standard; G7793) was prepared in PBST to generate glycerol standards. Glycerol standards, samples, and a PBST blank were then added to two microfuge tubes and the triglyceride reagent (Sigma; T2449) was added to one of the two tubes to free the glycerol backbone. All samples were incubated at 37 °C for 60 min. Samples were transferred to a clear, flat bottom 96-well plate and free glycerol reagent (Sigma; F6428) was added to each sample. Absorbance was measured at 540 nm. TAG concentration per sample was determined by subtracting the absorbance for the free glycerol in untreated samples from the total glycerol concentration in samples with the triglyceride reagent and then normalized to sample weight. TAG content in each sample is based on the triolein-equivalent standard curve.

### Lipid analysis

Lipogenesis and lipolysis were measured in adult flies by following the incorporation of radiolabeled sucrose^60^. Briefly, flies were fed with 5 µCi of ^14^C- sucrose incorporated into standard fly food for 24 h. After 24 h, half of the flies were collected and frozen at −80 °C to measure lipogenesis (0 h samples). The remaining flies were transferred to unlabeled standard fly food for 48 h and then immediately frozen at −80 °C to measure lipolysis (48 h samples). All flies were transferred into pre-weighed 1.7 ml Eppendorf tubes and then weighed to obtain fly mass. Frozen samples were homogenized with 200 µl of 2:1 chloroform: methanol mix for 15 min at room temperature and then spun at full speed for 15 min at room temperature. The supernatant was recovered and 40 µl of 0.9% (w/v) of NaCl was added. Samples were vortexed and spun gently to separate phases. The upper phase was removed, and the interphase was rinsed twice with 200 µl 1:1 methanol: water solution without mixing. The lower chloroform phase was used for scintillation counting.

### Starvation survival

Adult male flies (group housed, 20 flies/vial) on 1% (non-nutritional) agar. The percentage of flies alive was measured at least every 12 h.

### Feeding

Feeding was quantified in adult male flies using a capillary feeding assay^61^. Briefly, individual flies were placed into chambers (46 mm × 7 mm) containing 1% agar at the bottom and a glass capillary at the top. Each glass capillary contained a meniscus labeling dye to track food consumption and an aqueous food solution (2.5% sucrose + 2.5% yeast extract). All flies were habituated to the chamber and liquid diet 72 h before measuring food intake (glass capillaries were replaced daily). Total feeding was measured over 24 h and analyzed by Noah.py^41^.

### Statistical analysis and reproducibility

Normality of data was assessed with the D’Agostino-Pearson Test. Hypothesis testing was carried out with Student’s *t*-test (parametric), one/two-way ANOVA with post-hoc Sidak (parametric), Mann–Whitney (nonparametric), Kruskal–Wallis with post-hoc Dunn (nonparametric), or Kaplan–Meier with Mantel–Cox (Log-rank) tests. All statistical analyses were performed using GraphPad Prism 8.1.2 and Graphpad Prism 9.0.0. Immunohistochemistry experiment was performed in >5 nervous systems in 3 different experiments.

### Biological materials

Fly strains generated in this study are available upon request.

### Reporting summary

Further information on research design is available in the Nature Research Reporting Summary linked to this article.

## Supplementary information

Supplementary Information

Reporting summary

## Data Availability

The authors declare that the data supporting the findings of this study are available within the paper [and its Supplementary information]. Source data are provided with this paper.

## Source data

Source data
