# Supplementary material for: Neurofibromin regulates metabolic rate via neuronal mechanisms in Drosophila
Source: Nat Commun. 2021 Jul 13;12:4285. doi: 10.1038/s41467-021-24505-x (PMC8277851; doi:10.1038/s41467-021-24505-x)
Supplement: Supplementary file 1 — Supplementary Information [file 41467_2021_24505_MOESM1_ESM.pdf]

Supplementary Information

Neurofibromin regulates metabolic rate via neuronal mechanisms in *Drosophila*

Valentina Botero, Bethany A. Stanhope, Elizabeth B. Brown, Eliza C. Grenci, Tamara Boto, Scarlet J. Park, Lanikea B. King, Keith R. Murphy, Kenneth J. Colodner, James A. Walker, Alex C. Keene, William W. Ja, Seth M. Tomchik

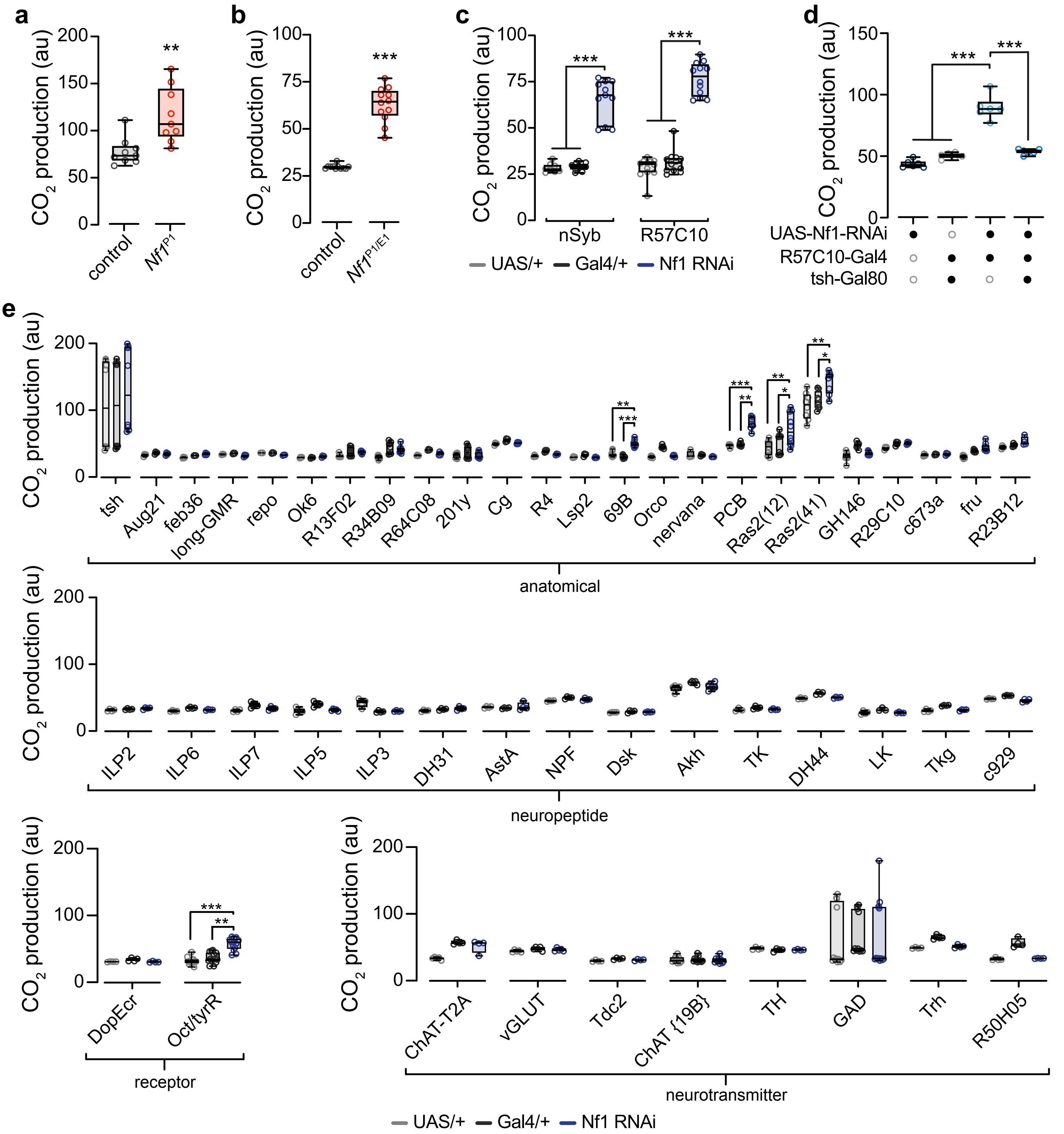

Supplementary Figure 1. Related to Figure 1 and Figure 2. **a.** CO_2 production in *Nf1*^{P1} mutants and w^{CS10} controls. **p = 0.002 (Mann-Whitney, two-sided n = 9). **b.** CO_2 production in *N1*$^{P1/E1}$ (n = 12) heteroallelic mutants compared to matched genetic background controls (n = 11). ***p = 0.0001 (Mann-Whitney, two-sided). **c.** CO_2 production when Nf1 was pan-neuronally knocked down with RNAi using Gal4 lines: nSyb- and R57C10-Gal4. Gal4/+ and UAS/+ are heterozygous controls. ***p<0.001 (Dunn's test, for nSyb-Gal4: n = 8 UAS-Nf1 RNAi/+, n = 10 nSyb-Gal4/+, n = 11 experimental group. For R57C10-Gal4: n = 12 per genotype). **d.** Pan-neuronal Nf1 knockdown with R57C10-Gal4, with and without the tsh-Gal80 repressor. ***p<0.001 (Sidak, two-sided n = 6). **e.** Raw data from the screen for neuronal subsets in which Nf1 knock down elevates CO_2 production, separated into four categories: anatomical, neuropeptides, receptors, and neurotransmitters. *p < 0.05, **p < 0.01, ***p < 0.001 (Dunn's test, two-sided; n = 7-14). au: arbitrary units. Exact sample sizes and p-values found in Source Data file for **e**. Each 'n' = single respirometer (containing 4 animals). Box plots – box: 1st to 3rd quartiles, median: line, whiskers: min-max, individual data points: circles.

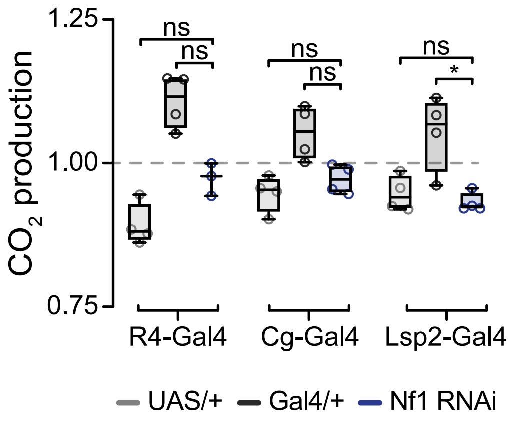

Supplementary Figure 2. Related to Figure 2.
CO_2 production, assayed by respirometry. Nf1 knockdown with RNAi driven by fat body specific Gal4 lines: R4-, Cg-, and Lsp2-Gal4. Gal4/+ and UAS/+ are heterozygous controls. For each driver, data are normalized to the mean of both controls. ns: not significant, (R4-Gal4, UAS- Nf1 RNAi experimental p = 0.39 re: R4-Gal4/+ ; p = 0.74 re: UAS-Nf1 RNAi/+ [Dunn's, two-sided n = 4 UAS-Nf1 RNAi/+, n = 4 R4-Gal4/+, n = 3 experimental group]), (Cg-Gal4, UAS-Nf1 RNAi experimental p = 0.07 re: Cg-Gal4/+; p >0.99 re: UAS-Nf1 RNAi/+ [Dunn's, two sided n = 4 per genotype]) (Lsp2-Gal4, Nf1 RNAi experimental *p = 0.02 re: Lsp2-Gal4/+; p >0.99 re: UAS-Nf1 RNAi/+ [Dunn's, two-sided n = 4 per genotype]). Each 'n' = single respirometer (containing 4 animals). Box plots – box: 1st to 3rd quartiles, median: line, whiskers: min-max, individual data points: circles.

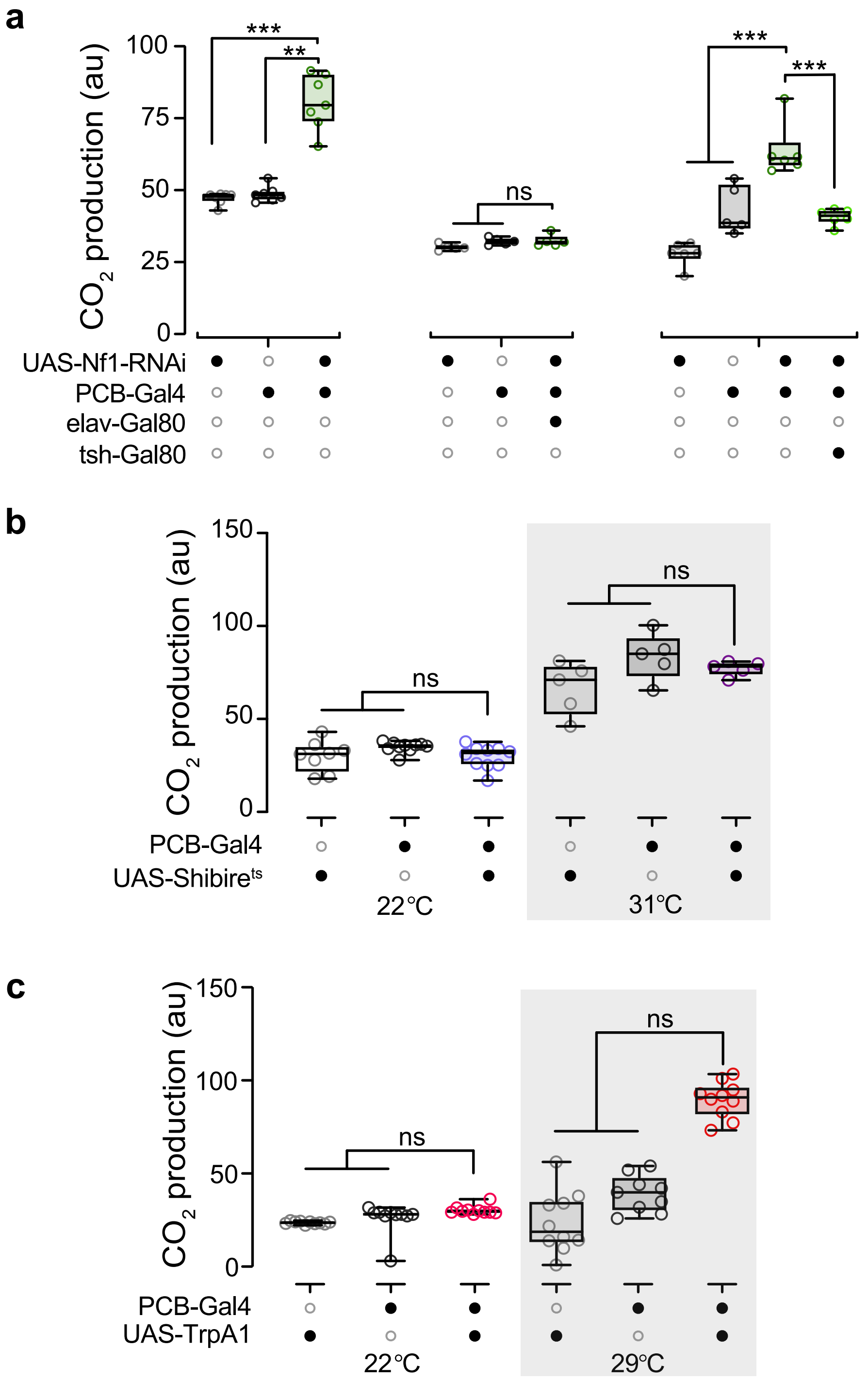

Supplementary Figure 3. Related to Figure 2. CO_2 production, assayed by respirometry.
a. Nf1 knockdown in neurons with PCB-Gal4 driver (left) (**p < 0.01 ***p < 0.001 [Dunn's test, two-sided n = 7]), with or without the elav-Gal80 repressor (middle) (experimental p = 0.068 re: UAS-Nf1 RNAi/+, p >0.999 re: PCB-Gal4/+ [Dunn's test, two-sided n = 5]), and with or without the tsh-Gal80 repressor (right) (***p < 0.001 [Sidak, two-sided n = 6]). **b.** CO_2 production assayed by respirometry when PCB-Gal4 neurons are blocked using UAS-Shibire^{ts} at 31 °C (right) and at control temperature (22 °C, left). At 22 °C experimental p >0.999 re: UAS-Shibire$^{t.s}$/+, p = 0.474 re: PCB-Gal4/+. (Sidak, two-sided n = 8 UAS-Shibire$^{t.s}$/+, n = 10 PCB-Gal4/+, and n = 10 experimental. At 31 °C experimental p = 0.162 re: UAS-Shibire^{ts}/+, p = 0.634 re: PCB-Gal4/+. (Sidak, two-sided n = 5 per genotype). ns: not significant. **c.** Normalized CO_2 production following activation of PCB-Gal4 neurons using UAS-TrpA1 at 29 °C (right) and at control temperature (22 °C, left). At 22 °C experimental p = 0.402 re: UAS-TrpA1/+, p = 0.784 re: PCB-Gal4/+. ns: not significant (Sidak, two-sided n = 10 per genotype). At 29 °C ***p < 0.001 (Sidak, two-sided n = 10 UAS-TrpA1/+, n = 9 PCB-Gal4/+, n = 10 experimental group). Each 'n' = single respirometer (containing 4 animals). Box plots – box: 1st to 3rd quartiles, median: line, whiskers: min-max, individual data points: circles.

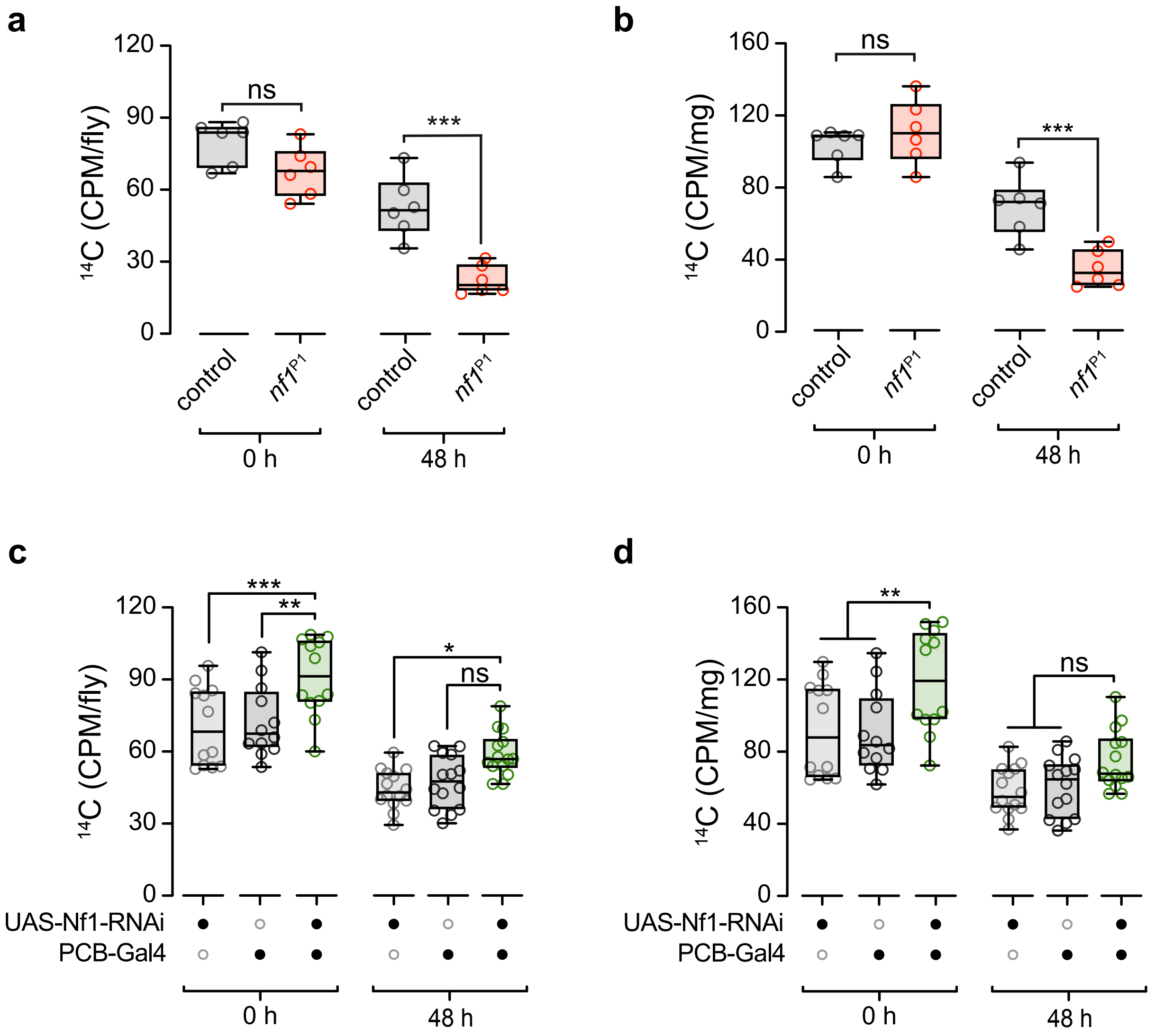

Supplementary Figure 4. Related to Figure 5.

a. Quantification of lipid turnover in *Nf1*^{P1} and *w*^{CS10} control flies via ^{14}C sucrose radiolabeling. Radioactivity measured as counts per minute (CPM) in a scintillator and normalized to number of flies. At time point zero hours (0 h) ns: not significant, p = 0.092, at 48 hours (48 h) ***p < 0.001, (Sidak, two-sided; n = 6 for both genotypes at both time points). **b.** Radioactivity measured as CPM and normalized to fly weight (mg). Data repeated from Figure 5f and from panel a here. At time point zero hours (0 h) ns: not significant, p = 0.604. at 48 hours (48 h) ***p < 0.001, (Sidak, two-sided; n = 6 for both genotypes at both time points). **c.** Quantification of lipid turnover in PCB Nf1 RNAi and controls. Radioactivity measured as counts per minute (CPM) in a scintillator and normalized to number of flies. At time point zero hours (0 h) experimental **p = 0.001 re: UAS-Nf1 RNAi/+, ** p = 0.003 re: PCB-Gal4/+. At 48 hours (48 h) experimental p = 0.016 re: UAS-Nf1 RNAi, p = 0.079 re: PCB-Gal4/+. ns: not significant, (Sidak, two-sided; n = 12 at 0 hour, n = 14 at 48 hours). **d.** Radioactivity measured as CPM and normalized to fly weight (mg). Data repeated from Figure 5g and from panel c here. At time point zero hours (0 h) experimental **p = 0.010 re: UAS-Nf1 RNAi/+, ** p = 0.006 re: PCB-Gal4/+. At 48 hours (48 h) experimental p = 0.129 re: UAS-Nf1 RNAi, p = 0.252 re: PCB-Gal4/+. ns: not significant, (Sidak, two-sided; n = 12 at 0 hour, n = 14 at 48 hours). Each 'n' represents a sample containing twenty animals. Box plots – box: 1st to 3rd quartiles, median: line, whiskers: min-max, individual data points: circles.
