# Supplementary material for: Neurofibromin regulates metabolic rate via neuronal mechanisms in Drosophila
Source: Nat Commun. 2021 Jul 13;12:4285. doi: 10.1038/s41467-021-24505-x (PMC8277851; doi:10.1038/s41467-021-24505-x)
Supplement: Supplementary file 2 — Reporting summary [file 41467_2021_24505_MOESM2_ESM.pdf]

nature research

Corresponding author(s): Tomchik, Seth M.

Last updated by author(s): May 17, 2021

Reporting Summary

Nature Research wishes to improve the reproducibility of the work that we publish. This form provides structure for consistency and transparency in reporting. For further information on Nature Research policies, see our Editorial Policies and the Editorial Policy Checklist.

Statistics

For all statistical analyses, confirm that the following items are present in the figure legend, table legend, main text, or Methods section.

n/a	Confirmed	
☐	☒	The exact sample size (*n*) for each experimental group/condition, given as a discrete number and unit of measurement
☐	☒	A statement on whether measurements were taken from distinct samples or whether the same sample was measured repeatedly
☐	☒	The statistical test(s) used AND whether they are one- or two-sided<br>*Only common tests should be described solely by name; describe more complex techniques in the Methods section.*
☒	☐	A description of all covariates tested
☐	☒	A description of any assumptions or corrections, such as tests of normality and adjustment for multiple comparisons
☐	☒	A full description of the statistical parameters including central tendency (e.g. means) or other basic estimates (e.g. regression coefficient) AND variation (e.g. standard deviation) or associated estimates of uncertainty (e.g. confidence intervals)
☐	☒	For null hypothesis testing, the test statistic (e.g. *F*, *t*, *r*) with confidence intervals, effect sizes, degrees of freedom and *P* value noted<br>*Give P values as exact values whenever suitable.*
☒	☐	For Bayesian analysis, information on the choice of priors and Markov chain Monte Carlo settings
☒	☐	For hierarchical and complex designs, identification of the appropriate level for tests and full reporting of outcomes
☒	☐	Estimates of effect sizes (e.g. Cohen's *d*, Pearson's *r*), indicating how they were calculated

Our web collection on statistics for biologists contains articles on many of the points above.

Software and code

Policy information about availability of computer code

Data collection	PhenoCapture 3.3 was used to collect sequential images of the liquid menicus position during respirometry experiments. Grooming videoswere collected using the Image Acquisition Toolbox in Matlab and saved as lossless Motion JPEG 2000 files. Confocal images were acquires on Leica TCS SP8 confocal microscopes with LAS X software.
Data analysis	Data were analyzed using Fiji version 2.0. Statistical analyses were performed with Graphpad Prism 9.0.0. Grooming was analyzed by watching videos frame-by-frame in Matlab.

For manuscripts utilizing custom algorithms or software that are central to the research but not yet described in published literature, software must be made available to editors and reviewers. We strongly encourage code deposition in a community repository (e.g. GitHub). See the Nature Research guidelines for submitting code & software for further information.

Data

Policy information about availability of data

All manuscripts must include a data availability statement. This statement should provide the following information, where applicable:

- Accession codes, unique identifiers, or web links for publicly available datasets
- A list of figures that have associated raw data
- A description of any restrictions on data availability

Figures contain individual data points. Data supporting the findings of this study are available within the paper and its supplementary information files. Raw data are available upon request and will be provided with no restrictions.

April 2020

Field-specific reporting

Please select the one below that is the best fit for your research. If you are not sure, read the appropriate sections before making your selection.

[x] Life sciences [ ] Behavioural & social sciences [ ] Ecological, evolutionary & environmental sciences

For a reference copy of the document with all sections, see nature.com/documents/nr-reporting-summary-flat.pdf

Life sciences study design

All studies must disclose on these points even when the disclosure is negative.

Sample size	Sample sizes were determined via reference to prior studies using equivalent techniques. (Respirometry - Yatsenko et al., 2014 and Van Voorhies et al., 2008; Grooming - King et al., 2020; Radiolabeling - Katewa et al., 2012; TAG - Gronke et al., 2003 and Wat et al., 2020; Feeding - Ja et al., 2007; Survival - Tong et al., 2007)
Data exclusions	No data were excluded from the study.
Replication	Major experimental findings were replicated multiple times on different days using different respirometers. All positive results from respirometry experiments were replicated once more on different days. All attempts at replication were successful.
Randomization	Flies were collected randomly from vials and assigned randomly to experimental or control groups where relevant. Experimental and control genotypes were run in parallel to control for extraneous variables (e.g., time of day effects).
Blinding	Blinding was performed in experiments that required manual scoring (grooming). In respirometry experiments, the genotypes were spatially interleaved in individual tubes across the experimental apparatus (to control for position effects), precluding blinding. In this case, the measurement was quantitative (meniscus position relative to a reference position), reducing the risk of bias.

Reporting for specific materials, systems and methods

We require information from authors about some types of materials, experimental systems and methods used in many studies. Here, indicate whether each material, system or method listed is relevant to your study. If you are not sure if a list item applies to your research, read the appropriate section before selecting a response.

Materials & experimental systems

n/a	Involved in the study	
[ ]	[x]	Antibodies
[x]	[ ]	Eukaryotic cell lines
[x]	[ ]	Palaeontology and archaeology
[ ]	[x]	Animals and other organisms
[x]	[ ]	Human research participants
[x]	[ ]	Clinical data
[x]	[ ]	Dual use research of concern

Methods

n/a	Involved in the study	
[x]	[ ]	ChIP-seq
[x]	[ ]	Flow cytometry
[x]	[ ]	MRI-based neuroimaging

Antibodies

Antibodies used	PRIMARY ANTIBODIES<br>• Rabbit anti-GFP (Invitrogen, A11122, Lot #2015993 and #20832201)<br>• Mouse anti-Brp (Developmental Studies Hybridoma Bank, NC82, 10/4/18)<br>• Mouse anti-Nf1 (mAb21): The et al., 1997 (ref in manuscript), additional information is not available<br>• Mouse anti-phospho-ERK (Sigma, M8159, Lot #057M4752V)<br>• Rabbit total ERK (rabbit, CST 9102, Lot #27)<br>• Mouse anti-β-tubulin (Developmental Studies Hybridoma Bank, E7, 11/29/18)<br>SECONDARY ANTIBODIES<br>• Goat anti-rabbit IgG (Alexa 488, Invitrogen, A11008, Lot #188524)<br>• Goat anti-mouse IgG (Alexa 633, Invitrogen, A21052, Lot #1848436)<br>• Goat anti-mouse IgG (Alexa 800, Invitrogen, A32730, Lot# UC279294)<br>• Donkey anti-rabbit IgG (Alexa 680, Invitrogen, A10043, Lot# 2044851)<br>• Horseradish peroxidase-conjugated anti-Rabbit IgG (H+L) (Jackson ImmunoResearch, 711035152)<br>• Horseradish peroxidase-conjugated anti-Mouse IgG (H+L) (Jackson ImmunoResearch, 715035151)
Validation	• Rabbit anti-GFP (Invitrogen A-11122): Western Blot and Immunostaining- Published species (Thermofisher Sci website, under product specification)

• Mouse anti-Brp (Developmental Studies Hybridoma Bank NC82): Immunostaining- Positive Tested Species Reactivity (DSHB website, under catalog fields)
• Mouse anti-Nfl (mAb21): IP and Western Blot - The et al., 1997

Animals and other organisms

Policy information about studies involving animals; ARRIVE guidelines recommended for reporting animal research

Laboratory animals	Male Drosophila melanogaster under 10 days old were used for this study.
Wild animals	Study did not involve wild animals.
Field-collected samples	Study did not involve samples collected from the field.
Ethics oversight	No ethical approval or guidance was required for the use of Drosophila.

Note that full information on the approval of the study protocol must also be provided in the manuscript.
